# Genotype-guided versus traditional clinical dosing of warfarin in patients of Asian ancestry: a randomized controlled trial

**DOI:** 10.1186/s12916-018-1093-8

**Published:** 2018-07-10

**Authors:** Nicholas L. Syn, Andrea Li-Ann Wong, Soo-Chin Lee, Hock-Luen Teoh, James Wei Luen Yip, Raymond CS Seet, Wee Tiong Yeo, William Kristanto, Ping-Chong Bee, LM Poon, Patrick Marban, Tuck Seng Wu, Michael D. Winther, Liam R. Brunham, Richie Soong, Bee-Choo Tai, Boon-Cher Goh

**Affiliations:** 1 Department of Haematology-Oncology, National University Cancer Institute, Singapore, Singapore; 2 Cancer Science Institute of Singapore, National University of Singapore, Singapore, Singapore; 3 Division of Neurology, Department of Medicine, National University Health System, Singapore, Singapore; 4 Department of Cardiology, National University Heart Centre, Singapore, Singapore; 5 Department of Medicine, University of Malaya Medical Centre, Kuala Lumpur, Malaysia; 6 Department of Pharmacy, National University Hospital, Singapore, Singapore; 7 Genome Institute of Singapore, Agency for Science, Technology and Research, Singapore, Singapore; 8 Translational Laboratory in Genetic Medicine, Agency for Science, Technology and Research, Singapore, Singapore; 9 Department of Medicine, Centre for Heart Lung Innovation, University of British Columbia, Vancouver, Canada; 10 Department of Pathology, Yong Loo Lin School of Medicine, National University Health System, Singapore, Singapore; 11 Saw Swee Hock School of Public Health, National University of Singapore, Singapore, Singapore; 12 Department of Pharmacology, Yong Loo Lin School of Medicine, National University Health System, Singapore, 119228 Singapore; 13 Department of Medicine, Yong Loo Lin School of Medicine, National University of Singapore, Singapore, Singapore

**Keywords:** Pharmacogenetics, Pharmacogenomics, Precision medicine, CYP2C9, Cytochrome P450, VKORC1, Warfarin, Anticoagulants, Anticoagulation, Polymorphism

## Abstract

**Background:**

Genotype-guided warfarin dosing has been shown in some randomized trials to improve anticoagulation outcomes in individuals of European ancestry, yet its utility in Asian patients remains unresolved.

**Methods:**

An open-label, non-inferiority, 1:1 randomized trial was conducted at three academic hospitals in South East Asia, involving 322 ethnically diverse patients newly indicated for warfarin (NCT00700895). Clinical follow-up was 90 days. The primary efficacy measure was the number of dose titrations within the first 2 weeks of therapy, with a mean non-inferiority margin of 0.5 over the first 14 days of therapy.

**Results:**

Among 322 randomized patients, 269 were evaluable for the primary endpoint. Compared with traditional dosing, the genotype-guided group required fewer dose titrations during the first 2 weeks (1.77 vs. 2.93, difference −1.16, 90% CI −1.48 to −0.84, *P* < 0.001 for both non-inferiority and superiority). The percentage of time within the therapeutic range over 3 months and median time to stable international normalized ratio (INR) did not differ between the genotype-guided and traditional dosing groups. The frequency of dose titrations (incidence rate ratio 0.76, 95% CI 0.67 to 0.86, *P* = 0.001), but not frequency of INR measurements, was lower at 1, 2, and 3 months in the genotype-guided group. The proportions of patients who experienced minor or major bleeding, recurrent venous thromboembolism, or out-of-range INR did not differ between both arms. For predicting maintenance doses, the pharmacogenetic algorithm achieved an *R*^*2*^ = 42.4% (*P* < 0.001) and mean percentage error of −7.4%.

**Conclusions:**

Among Asian adults commencing warfarin therapy, a pharmacogenetic algorithm meets criteria for both non-inferiority and superiority in reducing dose titrations compared with a traditional dosing approach, and performs well in prediction of actual maintenance doses. These findings imply that clinicians may consider applying a pharmacogenetic algorithm to personalize initial warfarin dosages in Asian patients.

**Trial registration:**

ClinicalTrials.gov NCT00700895. Registered on June 19, 2008.

**Electronic supplementary material:**

The online version of this article (10.1186/s12916-018-1093-8) contains supplementary material, which is available to authorized users.

## Background

While effective in preventing thromboembolic events, clinical application of warfarin is characterized by a narrow therapeutic index and often requires multiple dose titrations especially during the first few weeks of therapy. Well-managed warfarin therapy is associated with a reduction in the risk of complications [1], yet the majority of patients do not achieve long-term stable international normalized ratio (INR) within the therapeutic range [2], indicating the difficulty in identifying an optimal maintenance dose for individual patients. A growing body of evidence has emerged indicating that the cytochrome P450 2C9 (*CYP2C9*) and Vitamin K epoxide reductase complex subunit 1 (*VKORC1*) genotypes are associated with maintenance dose requirements, accounting for up to 40–45% of the inter-individual variability, depending on the populations and specific polymorphisms studied [3–5]. Accordingly, since 2007, the United States Food and Drug Administration product label for warfarin has been updated to reflect the potential value of incorporating genetic information into dose selection. Most major contemporary clinical trials and meta-analyses comparing genotype-guided dosing to routine clinical practice or clinically guided algorithms have employed surrogate outcomes and were not powered to demonstrate a difference in clinical endpoints [6–13].

To date, the utility of genotype-guided dosing remains unresolved, particularly in Asian populations, since most randomized studies have thus far been performed in predominantly Caucasian cohorts. Variation in the epidemiology of *VKORC1* and *CYP2C9* genetic polymorphisms across different ancestral populations could impact the performance of pharmacogenetically tailored dosing strategies [6, 7, 14]. The *VKORC1* H1/H1 haplotype, which confers high sensitivity to warfarin, is present in 74%, 42%, and 7% of self-identified Chinese, Malay, and Indian patients, respectively, while the CYP2C9*3 allele, which is associated with the poor metabolizer phenotype, is present in 7%, 9%, and 18% of patients, respectively [15]. On average, Asian patients homozygous for less-sensitive *VKORC1* haplotypes (H7, H8, or H9) and wild-type for *CYP2C9* will require more than 3.5 times the maintenance dosage needed by patients with the *VKORC1* H1/H1 haplotype and a copy of the CYP2C9*3 allele [15], highlighting a potential pitfall of empirical dose initiation and titration. Consequently, the application of pharmacogenetics to provide tailored doses to patients of Asian ancestry is particularly compelling. Accordingly, this randomized trial was conducted to test whether a pharmacogenetically based dosing algorithm, which was developed from a racially diverse Asian cohort [16], is non-inferior to traditional clinical dosing.

## Methods

The ethics review committees at participating centers approved the study protocol (Additional file 1). The study was conducted in accordance with Good Clinical Practice guidelines, and patients provided written informed consent prior to enrollment. All serious adverse events were reported to the Domain Specific Review Board and the Medical Clinical Research Committee, Ministry of Health, in accordance with published guidelines. The study is registered at ClinicalTrials.gov (Identifier: NCT00700895).

### Study design

This open-label, non-inferiority, randomized trial was conducted in three large tertiary hospitals in South East Asia. Randomization was computer generated with a 1:1 allocation ratio, and patients were allocated to the treatment arms by means of sequentially numbered, opaque, and sealed envelopes.

Eligibility criteria were age 18 years or older, a new indication for long-term anticoagulation with warfarin, and transaminases less than three times the upper limit of normal and bilirubin within normal range. Exclusion criteria were uncontrolled hypertension, peptic ulcer disease, previous history of liver disease, malabsorption syndrome or chronic diarrheal conditions, or any other medical conditions deemed unfit for warfarin administration based on the clinical judgment of primary treating physicians. Patients were not allowed to start warfarin before enrolment in the study. Demographic, clinical, and laboratory measurements were collected at baseline. Patient genotypes were determined through pyrosequencing as previously described [15, 16], and data on race or ethnicity was self-reported.

### Intervention

The study intervention period comprised of a dose initiation period (first 3 days) and a dose adjustment period (remainder of study). All patients were initiated on low-molecular weight heparins at the point of randomization. The expected turnaround time for genotyping was 2 days and warfarin was initiated on the third day in both groups. Patients randomized to the genotype-guided dosing strategy received their tailored dose for 3 consecutive days. This was calculated using an algorithm which takes into account the presence of the CYP2C9*3 allele, *VKORC1* 381 genotype, age, and weight [16]. The *VKORC1* 381 T > C single nucleotide polymorphism is in complete linkage disequilibrium with –1639G > A, and has been shown to discriminate the H1 and H7 haplotypes in Asian individuals [15, 16]. If genotype results were unavailable by the first scheduled dose of warfarin (day 1), the patient would be treated with the traditional dosing approach. Patients randomized to the traditional dosing approach were initiated using a standardized loading dose regimen used by the National University Hospital Anticoagulation Clinic consisting of per os warfarin 5 mg on days 1 and 2, followed by 3 mg on day 3. If the patient was more than 75 years of age, the dose on day 2 was lowered to 4 mg (Additional file 1). To account for instances when a different dose than that pre-specified was administered during the warfarin initiation period, deviations from the protocol-specified dose were considered as dose adjustments. During the first 14 days, there were three mandatory INR checks on day 6, between days 7 and 9, and between days 12 and 14. Based on these INR measurements, warfarin dose titrations in both groups as well as decisions to stop low-molecular weight heparin treatment were made according to usual clinical practice and centralized at the anticoagulation clinics (Additional file 1: Appendices 1 and 2). Included patients were followed up until day 90 after warfarin initiation. The number and frequency of follow-up visits were according to dosing tables that simulate real-world clinical practice (Additional file 1). If urgent anticoagulation was needed, patients on both study arms received low molecular weight heparin till INR reached the therapeutic range of 1.9 to 3.1 to avoid warfarin-induced thrombosis due to inhibition of Protein S and C. To avoid variability from different warfarin sources, Marevan^®^ tablets supplied by GlaxoSmithKline (Douglas Manufacturing Ltd., AK, NZ) were used throughout this study.

### Outcomes

The primary outcome was the number of dose titrations performed up to end of week 2 (day 14). Patients censored prior to day 14 were excluded from the modified intention-to-treat set due to insufficient data on dose titrations, INR, and other anticoagulation parameters for the evaluation of primary and secondary endpoints.

Secondary outcomes were time to stable INR, defined as the number of days from warfarin initiation to attaining therapeutic INR (≥ 1.9 and ≤ 3.1) for the latter of two consecutive measurements that are at least 7 days apart; percentage of time spent within the therapeutic range (PTTR), which was estimated using the linear interpolation method of Rosendaal et al. [17]; incidence of dose adjustments and INR monitoring during follow-up; and the proportions of patients who had a bleeding episode (classified as minor or major [18]), recurrent venous thromboembolism, and any measured INR value < 1.9 or > 3.1. The PTTR was included in June 2016 as a secondary outcome by way of protocol amendment following a meeting with the Scientific Review Committee for the Surveillance and Pharmacogenomics Initiative for Adverse Drug Reactions (SAPhIRE) program, who recommended that reporting of this endpoint would facilitate between-trial comparisons and enable meta-analyses of similar trials.

### Statistical analysis

The trial was powered to establish whether genotype-guided warfarin dose administration was non-inferior to traditional clinical dosing for the primary endpoint of number of dose titrations within the first 2 weeks of therapy. Based on previous data [19], the sample size was estimated assuming a conservative between-group mean difference of 1.0 and a common standard deviation of 1.4 dose titrations. Therefore, with 80% power and a one-sided type I error of 5%, a sample size of 270 would be able to demonstrate non-inferiority of the genotype-guided group for a predefined non-inferiority margin of 0.5 dose titrations. Assuming up to 15% loss to follow-up before day 14, a minimum of 320 patients was deemed necessary. If the upper bound of the 90% confidence interval (CI) of the difference in treatment (genotype-guided vs. traditional dosing) was lesser than 0.5, the null hypothesis would be rejected, which would signify that the genotype-guided strategy was non-inferior to the traditional dosing approach. When non-inferiority was proven, a two-tailed *t* test with an alpha value of 0.05 was used for superiority testing.

All other secondary endpoints were tests of superiority of genotype-guided dosing versus traditional dosing, and significance was defined as a two-tailed nominal *P* < 0.05. Time to stable INR was evaluated using the Kaplan–Meier method, and the log-rank test was used to compare differences. Percentage of time within the therapeutic range was compared using two-sample *t* tests. Mixed effects Poisson regression models were used to estimate incidence rate ratios (IRRs) for comparing the number of dose adjustments and INR measurements between interventions, while accounting for possible intra-subject correlation of count data which were measured at 1, 2, and 3 months. To account for the reduced follow-up time among patients who withdrew or discontinued the trial before day 90, we used an exposure variable in the Poisson regression for the number of days on trial. Predicted incidences of dose adjustments and INR measurements were estimated via Stata’s post-estimation command, immediately after fitting Poisson regression models. Differences in the proportions of patients who experienced minor or major bleeding, recurrent venous thromboembolism, and INR < 1.9 or > 3.1 were quantified using relative risks, with *P* values provided by Fisher’s exact test, and 95% CIs obtained from exact binomial distributions. Finally, the performance of the genotype-guided warfarin dosing model was evaluated using the Pearson’s product-moment correlation, with 95% CIs computed based on Fisher’s transformation, mean percentage error, root mean squared error, and Bland–Altman analysis.

All analyses were performed on a modified intention-to-treat basis and without imputation. Statistical analyses were performed in Stata version 13.0 (STATA Corp., College Station, TX, USA).

## Results

**Fig. 1:**
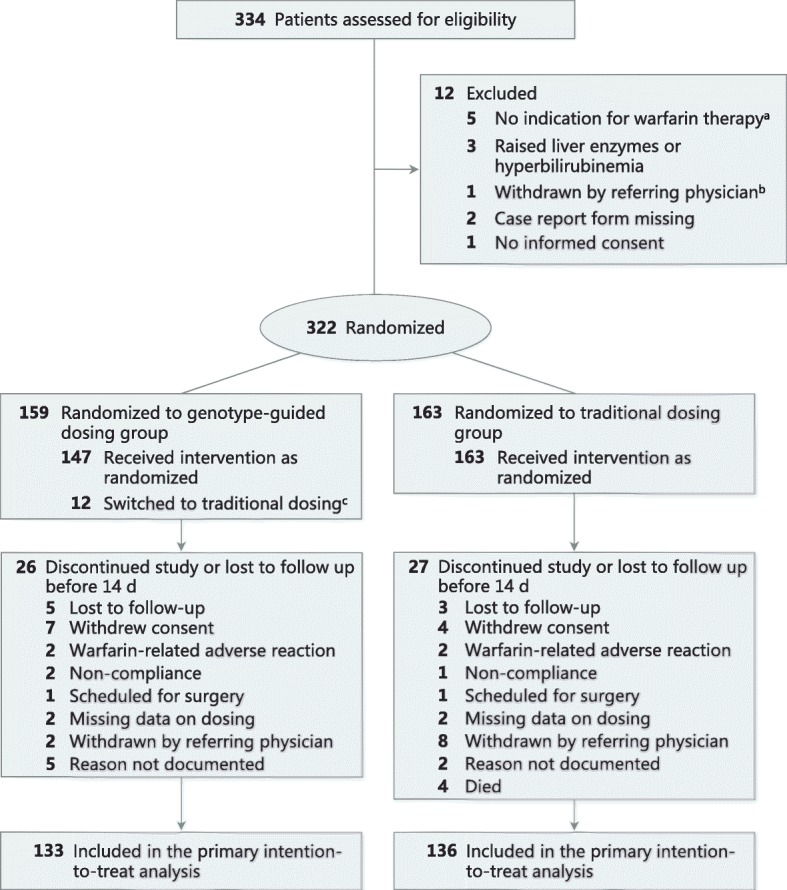
Flow of participants through the study of genotype-guided versus traditional-dosing of warfarin. ^a^Further tests were negative for thrombus. ^b^Potential drug interaction with concomitant corticosteroid medications. ^c^Patients were started on conventional dose of warfarin while awaiting genotype results

From May 11, 2007, through July 14, 2016, a total of 334 patients were screened, of whom 322 were randomized (159 to the genotype-guided group and 163 to the traditional dosing group) (Fig. 1). Baseline characteristics and genotypic distributions were well-balanced between both groups. Patients had a median age of 60 years (range, 19–89), and the majority of patients were male (58.4%) and of Chinese race (61.2%) (Table 1). Genotype results were available within the first 4 days for 147 of 159 (92.5%) patients randomized to the pharmacogenetics arm, and therefore these patients successfully received the first genotype-tailored dose on days 3 or 4 as scheduled in the protocol. Specifically, 88 (55.3%), 34 (21.4%), 14 (8.8%), and 11 (6.9%) patients had genotype results returned on days 1 through 4, respectively. The remaining 12 patients (7.5%) randomized to the pharmacogenetics arm were switched to traditional dosing as genotype results were not available by day 5.

**Table 1:** Baseline clinical characteristics and demographics

	Traditional dosing (*n* = 163)	Genotype-guided dosing (*n* = 159)
Age, mean (SD), years	59.4 (14.5)	58.4 (14.3)
Male, No. (%)	88 (54.0)	100 (62.9)
Weight, mean (SD), kg	66.9 (16.8)	67.3 (14.1)
Race, No. (%)		
Chinese	98 (60.1)	99 (62.3)
Malay	39 (23.9)	32 (20.1)
Indian	17 (10.4)	14 (8.8)
Others	9 (5.5)	14 (8.8)
*CYP2C9* genotype, No./total (%)		
Presence of *3 allele	11/160 (6.9)	7/158 (4.4)
*VKORC1*–381 genotype, No./total (%)		
C/C	91/162 (56.2)	97/159 (61.0)
C/T	47/162 (29.0)	43/159 (27.0)
T/T	24/162 (14.8)	19/159 (12.0)
Indication, No./total (%)		
Atrial fibrillation	55/160 (34.4)	61/156 (39.1)
Stroke	11/160 (6.9)	11/156 (7.1)
Deep vein thrombosis	44/160 (27.5)	42/156 (26.9)
Pulmonary embolism	19/160 (11.9)	17/156 (10.9)
Left ventricular thrombus	17/160 (10.6)	18/156 (11.5)
Others	26/160 (16.3)	14/156 (9.0)
Amiodarone, No./total (%)	3/159 (1.9)	7/156 (4.5)
Low-molecular weight heparins, No./total (%)	78/159 (49.1)	88/157 (56.1)
Medical history, No./total (%)		
Stroke	16/160 (10.0)	10/157 (6.4)
Deep vein thrombosis	7/160 (4.4)	4/157 (2.6)
Pulmonary embolism	2/160 (1.3)	3/157 (1.9)
Myocardial infarction	8/160 (5.0)	17/157 (10.8)
Congestive heart failure	21/160 (13.1)	18/157 (11.5)
Hypertension	86/160 (53.8)	92/157 (58.6)
Type 2 diabetes mellitus	58/160 (36.3)	56/157 (35.7)
Centre, No. (%)		
National University Hospital, Singapore	144 (88.3)	144 (90.6)
University of Malaya Medical Centre, Malaysia	15 (9.2)	15 (9.4)
Tan Tock Seng Hospital, Singapore	4 (2.5)	0 (0.0)

In the primary analysis only patients who received warfarin treatment for at least 14 days were included. Thus, 133 (83.6%) and 136 (83.4%) patients from the genotype-guided and traditional dosing groups, respectively, were included in the modified intention-to-treat set (reasons for censoring are shown in Fig. 1). Clinical demographics and genotypic frequencies among patients who discontinued warfarin before 14 days of therapy are detailed in Additional file 2: Table S1, and baseline characteristics were relatively similar as compared to the overall population. The causes of death of four patients in the traditional dosing group were cardiac arrest, retroperitoneal bleed, hospital-associated pneumonia, and advanced cancer. Median duration of warfarin therapy was comparable between the two groups, and was 90.0 days (interquartile range (IQR) 83.8–90.0 days) in the traditional dosing group and 90.0 days (IQR 77.0–90.0 days) in the genotype-guided group.

### Primary outcome

**Fig. 2:**
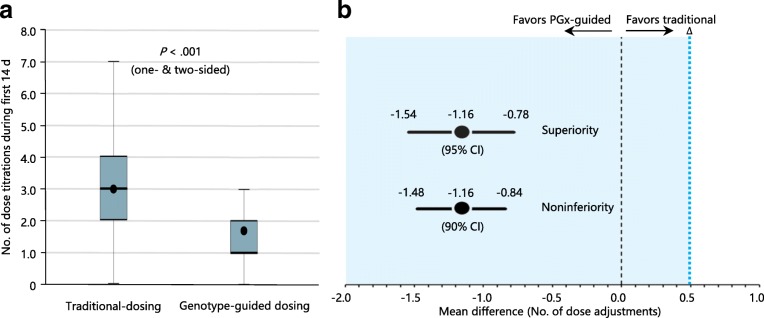
**a** Number of dose titrations within first 2 weeks of therapy. Dark horizontal lines indicate median values. The circle represents the mean. The top line of the box indicates the 75th percentile, and the bottom line of the box indicates the 25th percentile. The top and bottom whiskers indicate the 97.5th and 2.5th percentiles, respectively. **b** Non-inferiority and superiority comparison for the primary endpoint of mean difference in number of dose titrations within first 2 weeks of therapy. Error bars indicate two-sided 90% or 95% CI, respectively. Since the upper bound of the 90% CI of the difference in treatment (genotype-guided vs. traditional dosing) was less than 0.5, the genotype-guided strategy was non-inferior to the traditional dosing approach. The upper bound of the 95% CI did not exceed 0, indicating that superiority was also demonstrated

The average number of dose titrations performed up to the 14th day was 1.77 (95% CI 1.55 to 2.00) in the genotype-guided group versus 2.93 (95% CI 2.63 to 3.24) in the traditional dosing group (mean difference −1.16, 90% CI −1.48 to −0.84). Thus, both non-inferiority (*P* < 0.001), according to the pre-specified definition, and superiority (*P* < 0.001) of the genotype-guided dosing algorithm over the traditional dosing algorithm was established (Fig. 2). This difference in mean number of dose titrations corresponds to an IRR of 0.60 (95% CI 0.51 to 0.70, two-sided *P* < 0.001) in favor of the genotype-guided dosing algorithm.

### Secondary outcomes

**Fig. 3:**
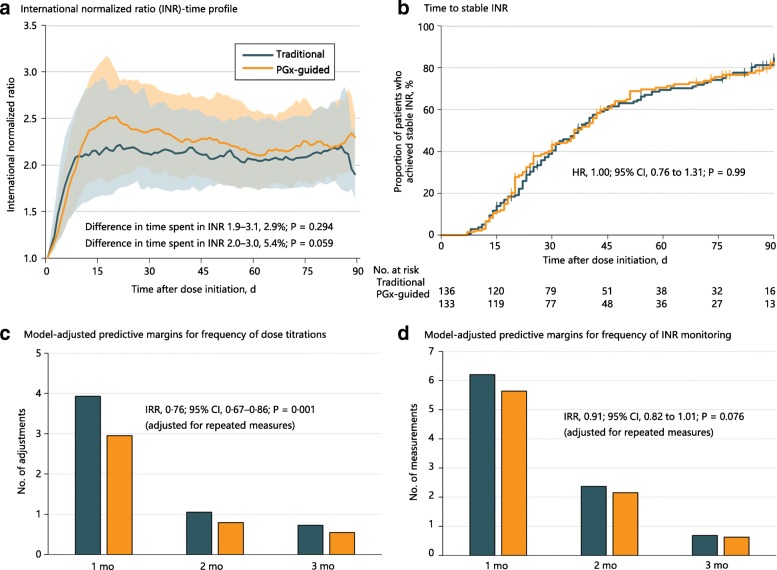
Secondary endpoints in the study. **a** Median international normalized ratio (INR) trajectory and 20–80th percentile bands over a 90-day period. **b** Kaplan–Meier failure functions for the proportion of patients who achieved stable INR, which was not significantly different between treatment groups. Spikes on the Kaplan–Meier curves represent censoring. **c** and **d** Number of dose titrations and INR monitoring at 1, 2, and 3 months, predicted using STATA’s post-estimation command

The effect of warfarin therapy on INR trajectories is depicted in Fig. 3a. The median time to stable INR, defined as the number of days from randomization to the latter of two consecutive measurements that are at least 7 days apart, was 36 days (IQR 20–74 days) in the genotype-guided group versus 37 days (IQR 22–76 days) in the traditional dosing group. A total of 103 (77.4%) patients in the genotype-guided group achieved stable INR as compared with 108 (79.4%) in the traditional dosing groups, and the rate of attaining stable INR was not statistically different between groups (genotype-guided vs. traditional dosing HR 1.00, 95% CI 0.76 to 1.31, *P* = 0.99) (Fig. 3b). There was no evidence of difference in the percentage of time in the therapeutic range (based on the pre-specified INR target of 1.9–3.1) over the follow-up period (Fig. 3a). The percentage of time in the pre-specified therapeutic range were 60.0% (95% CI 56.1% to 64.0%) in the genotype-guided group compared with 57.1% (95% CI 53.2% to 61.0%) in the traditional dosing group (mean difference 2.9%, 95% CI –2.6% to 8.4%, *P* = 0.29). Based on a post-hoc target INR range of 2.0–3.0, the percentage of time in the therapeutic range was 52.5% (95% CI 48.5% to 56.5%) in the genotype-guided group compared with 47.1% (95% CI 43.0% to 51.1%) in the traditional dosing group (mean difference 5.4%, 95% CI –0.2% to 11.1%, *P* = 0.059).

The number of dose adjustments and INR measurements generally decreased over the first through third months of treatment (Fig. 3c, d). The frequency of dose adjustments was significantly lower in the genotype-guided group over the entire duration of treatment (4.51 ± 2.20 vs. 6.06 ± 2.93, IRR 0.76, 95% CI 0.67 to 0.86, *P* = 0.001) compared to the traditional dosing group, after accounting for variation in between-individual exposure time and within-individual correlations in repeated measurements using a log-linear mixed effects Poisson model. The frequency of INR measurements did not differ significantly between the genotype-guided group versus the traditional dosing group over the follow-up period (8.63 ± 4.26 vs. 9.48 ± 4.05, IRR 0.91, 95% CI 0.82 to 1.01, *P* = 0.076).

Minor bleeding complications occurred in 8/132 (6.1%, 95% CI 2.7% to 11.6%) patients in the genotype-guided group versus 8/135 (5.9%, 95% CI 2.6% to 11.3%) patients in the traditional dosing group (RR 1.02, 95% CI 0.40 to 2.64, *P* = 0.96); major bleeding complications occurred in 5/133 (3.8%, 95% CI 1.2% to 8.6%) and 5/136 (3.7%, 95% CI 1.2% to 8.4%) patients, respectively (RR 1.02, 95% CI 0.30 to 3.45; *P* = 0.97); and recurrent venous thromboembolism was documented in 2/132 (1.5%, 95% CI 0.2% to 5.4%) and 1/135 (0.7%, 95% CI 0.02% to 4.1%) patients, respectively (RR 2.05, 95% CI 0.19 to 22.3, *P* = 0.55). Furthermore, an INR value of less than 1.9 was recorded at least once in 129/132 (97.7%, 95% CI 93.5% to 99.5%) in the genotype-guided group versus 128/135 (94.8%, 95% CI 89.6% to 97.9%) in the traditional dosing group (RR 1.03, 95% CI 0.98 to 1.08, *P* = 0.21), whereas a measured INR of greater than 3.1 occurred in 59/132 (44.7%, 95% CI 36.0% to 53.6%) and 60/135 (44.4%, 95% CI 35.9% to 53.2%), respectively (RR 1.01, 95% CI 0.77 to 1.31, *P* = 0.97). Thus, no significant differences in these safety outcomes were detected between the genotype-guided and traditional dosing regimens.

**Fig. 4:**
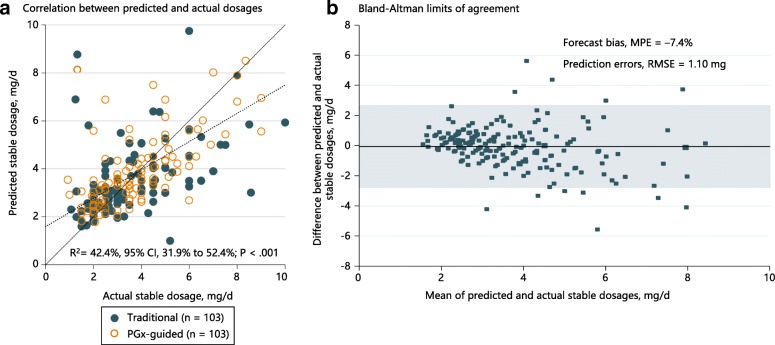
**a** Scatterplot of predicted versus actual maintenance dosage. The solid line indicates the line of equivalence, while the dashed line represents the linear fit between algorithm-predicted and actual maintenance warfarin dosages. The plot includes only patients who have achieved stable international normalized ratio (INR), which is defined as attaining therapeutic INR (≥ 1.9 and ≤ 3.1) for two consecutive measurements that are at least 7 days apart. **b** Bland–Altman assessment of pharmacogenetic dosing model’s predictive performance. Shaded area indicates 95% confidence limits

The predictive performance of the pharmacogenetic maintenance dose model was also evaluated (Fig. 4a). Based on available data, the predicted daily maintenance dosages correlated positively with actual documented stable dosages (*R*^*2*^ = 42.4%, 95% CI 31.9% to 52.4%, *P* < 0.001) with a root mean-squared error of 1.10 mg and a mean percentage error of −7.4% (Fig. 4b), indicating a low level of positive forecast bias and a respectable level of predictive accuracy.

## Discussion

Warfarin and its analogues have been used as oral anticoagulants for more than 60 years, and many institutions worldwide still employ an empirical dose initiation protocol despite the known inter-individual variability in dose requirements and anticoagulation outcomes. In this study involving patients with a new indication for warfarin therapy, the number of dose titrations in the first 2 weeks, and also throughout the follow-up period, was lower in the genotype-guided group than in the traditional dosing group. Furthermore, the genotype dosing algorithm accurately predicted the maintenance dose requirements in patients who achieved stable INR. Our findings are consistent with results from the COUMAGEN-I trial [10], which showed a similar advantage for accurate prediction of stable doses and frequency of dosing adjustments in the pharmacogenetically guided arm, but similar outcomes in terms of anticoagulation control parameters such as the fraction of out-of-range INRs.

The finding that percentage of time spent within the therapeutic range (PTTR) was not statistically different between the two groups was similar to that observed in the recent COAG trial [6], but different from that in the EU-PACT [7], COUMAGEN-II [8], and GIFT trials [13]. Although widely interpreted as failure of genotype-guided dosing, a major confounder when interpreting these endpoints are the incidence of dose adjustments and INR monitoring performed in the genotype-guided group and in the control group. Given that dosing titrations were performed more frequently in our control arm than in the genotype-guided arm, this could have inflated the PTTR in the traditional dosing group and diminished any apparent benefit of genotype-guided dosing. Notwithstanding, our trial was not designed to answer whether genotype-guided dosing improves anticoagulation control when controlled for the number of dose adjustments. Future trials may therefore consider incorporating this potential confounder as an adjustment or stratification variable into their statistical analysis plans.

Some investigators have advocated that warfarin dosing algorithms should be population specific and evaluated in populations similar to those from which they were developed [14, 20]. Therefore, although several genotype-based dosing algorithms have been proposed [21–25], a strength of this study is the selection of an algorithm [16] developed and validated in a cohort that is racially comparable to the current study population.

The use of clinical algorithms for dose initiation and dose adjustment were applied in the EU-PACT and COAG studies, which enrolled predominantly Caucasian and Black populations. These dosing algorithms have not been validated in Asian populations, and therefore were not used in this study. Moreover, the fact that PTTR of the control group was comparable between our study and the clinical algorithm dosing groups in the Western studies would suggest no difference in outcomes with application of clinical algorithm-based dosing in this study.

From the viewpoint of clinical applicability, the findings of this study are representative of and generalizable to an ethnogeographically diverse Asian population; the Chinese and Indian patients in these studies are mostly migrants from China and South India, and the Malay patients are indigenous to the islands of the Indonesian archipelago, including Malaya, Sumatra, and Java [15, 16]. Reduction of frequency of dose titrations (the primary endpoint) using genotype-based algorithms is highly desirable in the context of Asia, where long distances from rural or suburban areas to healthcare facilities poses a barrier to optimal anticoagulation therapy.

The use of a non-inferiority design in this study deserves mention. Firstly, no comparative data about the capability of a genotype-guided dosing strategy in reducing the number of dose titrations was available at the time of conceptualization, although early observational and retrospective studies suggested that genotyping may have value in informing dose selection [26]. Considering that, at the time this trial was conceived, there was no prospective data comparing pharmacogenetically guided dosing versus traditional dosing, it was arguably a reasonable concern that pharmacogenetically guided dosing could be worse than traditional dosing in terms of the number of dose adjustments required in the first 2 weeks. As such, a non-inferiority null hypothesis that genotype-guided dosing could be worse than traditional dosing was arguably justifiable and valid at that time. Therefore, the trial was designed to demonstrate that pharmacogenetically guided dosing was not less efficacious than conventional dosing, and as secondary endpoints, to test whether a genotype-guided algorithm accurately predicts maintenance dose requirements and improves other markers of anticoagulation control.

There are several limitations of this study. Firstly, we did not evaluate a combination of a loading-dose algorithm and dose-revision algorithm in the genotype-guided arm, which may be responsible for the PTTR advantage observed in the EU-PACT study [7]. Simulations integrating a pharmacokinetic-pharmacodynamic model [27, 28] and genotype frequencies present in a Han-Chinese cohort in fact suggests that the deployment of genetically informed loading doses and dose revisions is superior to a clinically guided dosing regimen [29]. Nevertheless, this study was not designed to test a difference in the PTTR, which as mentioned earlier, may be confounded by imbalance in the number of dose titrations performed in the two groups, nor was it powered to detect differences in the outcome of bleeding and re-thrombosis. Other limitations include the lack of adjustment for multiplicity among the secondary endpoints; its open-label design, which potentially introduces ascertainment bias; and a lack of pre-specified adjusted or subgroup analyses, for example, assessment of endpoints according to ethnic grouping, which may have afforded further information on the utility of genotype-guided warfarin dosing. Furthermore, approximately 16% of patients were excluded because they did not continue warfarin for 14 days, although it should be noted that this attrition rate is in line with the expected dropout rate of approximately 15% that was accounted for in our sample size calculations. Moreover, the study was designed as a multicenter clinical trial yet the majority (86.7%) of patients were enrolled at a single tertiary care center due to slow accrual in the other centers. This therefore limits the generalizability of our results.

## Conclusions

In this randomized, non-inferiority clinical trial that included 322 adults of South East Asian ancestry, genotype-guided dosing reduced the number of dose titrations during the first 2 weeks compared to traditional dosing (1.77 vs. 2.93) while maintaining similar INR time within therapeutic ranges. The reduction in frequency of dose revisions persisted over the 90-day follow-up period (incidence rate ratio 0.76). The genotype-guided algorithm also accurately predicted maintenance dose requirements. These findings imply that clinicians treating Asian patients may consider applying a pharmacogenetic algorithm to personalize initial warfarin dosages.

## Additional files

Additional file 1: Statistical Analysis Plan (SAP) and Study Protocol. (PDF 307 kb)

Additional file 2: **Table S1.** Baseline characteristics of patients excluded from primary analysis. (DOCX 25 kb)

## Abbreviations

CYP2C9: cytochrome P450 2C9
INR: International normalized ratio
PTTR: percentage of time spent in therapeutic range
VKORC1: Vitamin K epoxide reductase complex 1

## References

[1] Passman R (2016). Time in therapeutic range in warfarin-treated patients: is very good good enough?. JAMA.

[2] Pokorney SD, Simon DN, Thomas L, Gersh BJ, Hylek EM, Piccini JP (2016). Stability of international normalized ratios in patients taking long-term warfarin therapy. JAMA.

[3] Jonas DE, McLeod HL (2009). Genetic and clinical factors relating to warfarin dosing. Trends Pharmacol Sci.

[4] Johnson JA, Cavallari LH (2015). Warfarin pharmacogenetics. Trends Cardiovasc Med.

[5] Syn NL-X, Yong W-P, Lee S-C, Goh B-C (2015). Genetic factors affecting drug disposition in Asian cancer patients. Expert Opin Drug Metab Toxicol.

[6] Kimmel SE, French B, Kasner SE, Johnson JA, Anderson JL, Gage BF (2013). A pharmacogenetic versus a clinical algorithm for warfarin dosing. N Engl J Med.

[7] Pirmohamed M, Burnside G, Eriksson N, Jorgensen AL, Toh CH, Nicholson T (2013). A randomized trial of genotype-guided dosing of warfarin. N Engl J Med.

[8] Anderson JL, Horne BD, Stevens SM, Woller SC, Samuelson KM, Mansfield JW (2012). A randomized and clinical effectiveness trial comparing two pharmacogenetic algorithms and standard care for individualizing warfarin dosing (CoumaGen-II). Circulation.

[9] Brensinger CM, Kimmel SE (2011). Genetic warfarin dosing tables versus algorithms. JACC.

[10] Anderson JL, Horne BD, Stevens SM, Grove AS, Barton S, Nicholas ZP (2007). Randomized trial of genotype-guided versus standard warfarin dosing in patients initiating oral anticoagulation. Circulation.

[11] Stergiopoulos K, Brown DL (2014). Genotype-guided vs clinical dosing of warfarin and its analogues. JAMA Intern Med.

[12] Dahal K, Sharma SP, Fung E, Lee J, Moore JH, Unterborn JN (2015). Meta-analysis of randomized controlled trials of genotype-guided vs standard dosing of warfarin. Chest.

[13] Gage BF, Bass AR, Lin H, Woller SC, Stevens SM, Al-Hammadi N (2017). Effect of genotype-guided warfarin dosing on clinical events and anticoagulation control among patients undergoing hip or knee arthroplasty. JAMA.

[14] Limdi NA, Brown TM, Yan Q, Thigpen JL, Shendre A, Liu N (2015). Race influences warfarin dose changes associated with genetic factors. Blood.

[15] Lee S-C, Ng S-S, Oldenburg J, Chong P-Y, Rost S, Guo J-Y (2006). Interethnic variability of warfarin maintenance requirement is explained by VKORC1 genotype in an Asian population. Clin Pharmacol Ther.

[16] Tham L-S, Goh B-C, Nafziger A, Guo J-Y, Wang L-Z, Soong R (2006). A warfarin-dosing model in Asians that uses single-nucleotide polymorphisms in vitamin K epoxide reductase complex and cytochrome P450 2C9. Clin Pharmacol Ther.

[17] Rosendaal FR, Cannegieter SC, Van Der Meer FJM, Briet E (1993). A method to determine the optimal intensity of oral anticoagulant therapy. Thromb Haemost.

[18] Levine M, Gent M, Hirsh J, Leclerc J, Anderson D, Weitz J (1996). A comparison of low-molecular-weight heparin administered primarily at home with unfractionated heparin administered in the hospital for proximal deep-vein thrombosis. N Engl J Med.

[19] Kovacs MJ, Rodger M, Anderson DR, Morrow B, Kells G, Kovacs J (2003). Comparison of 10-mg and 5-mg warfarin initiation nomograms together with low-molecular-weight heparin for outpatient treatment of acute venous thromboembolism. A randomized, double-blind, controlled trial. Ann Intern Med.

[20] Kubo K, Ohara M, Tachikawa M, Cavallari LH, Lee MTM, Wen MS (2017). Population differences in S-warfarin pharmacokinetics among African Americans, Asians and whites: their influence on pharmacogenetic dosing algorithms. Pharmacogenomics J.

[21] Gage BF, Eby C, Milligan PE, Banet GA, Duncan JR, McLeod HL (2003). Use of pharmacogenetics and clinical factors to predict the maintenance dose of warfarin. Thromb Haemost.

[22] Sconce EA, Khan TI, Wynne HA, Avery P, Monkhouse L, King BP (2005). The impact of CYP2C9 and VKORC1 genetic polymorphism and patient characteristics upon warfarin dose requirements: proposal for a new dosing regimen. Blood.

[23] Klein TE, Altman RB, Eriksson N, Gage BF, Kimmel SE, Lee MT, Limdi NA, Page D, Roden DM, Wagner MJ, JJ CMD, International Warfarin Pharmacogenetics Consortium (2009). Estimation of the warfarin dose with clinical and pharmacogenetic data. N Engl J Med.

[24] Lenzini P, Wadelius M, Kimmel SE, Anderson JL (2010). Integration of genetic, clinical, and INR data to refine warfarin dosing. Clin Pharmacol Ther.

[25] Avery PJ, Jorgensen A, Hamberg AK, Wadelius M, Pirmohamed M, Kamali F (2011). A proposal for an individualized pharmacogenetics-based warfarin initiation dose regimen for patients commencing anticoagulation therapy. Clin Pharmacol Ther.

[26] Voora D, Eby C, Linder MW, Milligan PE, Bukaveckas BL, McLeod HL (2005). Prospective dosing of warfarin based on cytochrome P-450 2C9 genotype. Thromb Haemost.

[27] Hamberg a-K, Dahl M-L, Barban M, Scordo MG, Wadelius M, Pengo V (2007). A PK-PD model for predicting the impact of age, CYP2C9, and VKORC1 genotype on individualization of warfarin therapy. Clin Pharmacol Ther.

[28] Hamberg a-K, Wadelius M, Lindh JD, Dahl ML, Padrini R, Deloukas P (2010). A pharmacometric model describing the relationship between warfarin dose and INR response with respect to variations in CYP2C9, VKORC1, and age. Clin Pharmacol Ther.

[29] Syn NL-X, Lee S-C, Brunham LR, Goh B-C (2015). Pharmacogenetic versus clinical dosing of warfarin in individuals of Chinese and African-American ancestry: assessment using data simulation. Pharmacogenet Genomics.

