# Supplementary material for: Genotype-guided versus traditional clinical dosing of warfarin in patients of Asian ancestry: a randomized controlled trial
Source: BMC Med. 2018 Jul 10;16:104. doi: 10.1186/s12916-018-1093-8 (PMC6038204; doi:10.1186/s12916-018-1093-8)
Supplement: Supplementary file 1 — Statistical Analysis Plan (SAP) and Study Protocol. (PDF 307 kb) [file 12916_2018_1093_MOESM1_ESM.pdf]

Statistical Analysis Plan (SAP)

A Randomized Controlled Trial to Assess the Clinical Benefits of a Pharmacogenetics-Guided Dosing Regimen for Calculating Warfarin Maintenance Dose (Protocol No: PG01/11/06)

Table of Contents

1. Executive Summary **2**

2. Introduction **3**

2.1 Changes to Study Protocol 3

3. Study Objectives **3**

4. Study Design **3**

5. Sample Size Calculation **4**

6. Reporting of Patients Baseline Characteristics **4**

7. Study Endpoints **4**

7.1 Number of dose titrations performed up to days 12-14 4

7.2 Secondary Endpoints 4

7.2.1 Time to stable INR 5

7.2.2 Number of dose adjustments at 1, 2 and 3 months 5

7.2.3 Number of INR assessments at 1, 2 and 3 months 5

7.2.4 Proportion of patients whose INR exceeded 3.0 5

7.2.5 Frequency of bleeding episodes 5

7.2.6 Incidence of recurrent venous thromboembolism within 90 days of diagnosis 6

7.2.7 Percentage of time spent within therapeutic INR range 6

7.2.8 Accuracy and precision of the pharmacogenetic maintenance dose-prediction model 6

7. References **7**

Protocol Version and Date: Version 13, April 2014
SAP Version and Date: Version 1, June 2016

1. Executive Summary

**Title**	A Randomized Controlled Trial to Assess the Clinical Benefits of a Pharmacogenetics-Guided Dosing Regimen for Calculating Warfarin Maintenance Dose
**Sponsor**	National Medical Research Council
**Principal Investigator**	Boon-Cher Goh, M.D.
**Study Agent**	Warfarin (Marevan®)
**Study Design**	This is a multicentre, open-label, randomised (1:1) controlled trial which uses a non-inferiority study design.<br>Primary analysis will be based on intention-to-treat.
**Sample Size**	The sample size of 270 provides 80% power to detect a non-inferiority margin of 0.5 with a one-sided alpha of 0.05, given a between-group difference in mean number of dose titrations of 1.0 and common standard deviation of 1.4.<br>Assuming an attrition rate of 15%, 320 patients would be enrolled and randomly allocated 1:1 to a study group.
**Interim Analysis**	None
**Patients**	Eligible patients must be at least 18 years of age; have a new indication for warfarin; have no previous history of liver disease, malabsorption syndrome or chronic diarrheal conditions; do not have uncontrolled hypertension, peptic ulcer disease or any medical condition deemed unfit for warfarin therapy.<br>Written informed consent is required prior to enrolment.
**Primary Objective**	To compare pharmacogenetics-guided versus traditional dosing of warfarin in terms of anticoagulation control parameters
**Treatment Duration**	Patients will be followed for 3 months from treatment initiation
**Primary Endpoint**	1. Number of dose titrations performed up to days 12-14
**Secondary Endpoints**	2. Time to stable INR*<br>3. Number of dose adjustments at 1, 2 and 3 months<br>4. Number of INR assessments at 1, 2 and 3 months<br>5. Proportion of patients whose INR exceeded 3.0<br>6. Frequency of bleeding episodes<br>7. Incidence of recurrent venous thromboembolism with 90 days of diagnosis<br>8. Percentage of patients achieving target INR by days 12-14*<br>9. Percentage of time spent within therapeutic INR range*<br>10. Accuracy (MPE%) and precision (RMSE%) of the pharmacogenetic maintenance dose-prediction model<br>* These endpoints use target INR range >1.9 and ≤ 3.1
**Study Sites**	1. National University Hospital, Singapore<br>2. University of Malaya Medical Centre, Malaysia<br>3. Tan Tock Seng Hospital, Singapore
**Clinical Trial Registration Details**	The clinical trial is registered at ClinicalTrials.gov with the identifier NCT00700895.

2. Introduction

The Statistical Analysis Plan (SAP) provides a comprehensive and detailed description of the statistical design and methodologies, including pre-planned analyses for the study “A Randomized Controlled Trial to Assess the Clinical Benefits of a Pharmacogenetics-Guided Dosing Regimen for Calculating Warfarin Maintenance Dose” (Protocol No: PG01/11/06).

2.1 Changes to Study Protocol

The SAP summarises the statistical design and methodologies presented in the April 2014 version of the study protocol; and where differences exist, supersedes the study protocol. Any changes to the study protocol as listed in this SAP have been submitted to and approved by the Domain Specific Review Board (DSRB) of the National Healthcare Group (NHG). Any differences with the study protocol are clearly described, and the rationale for the amendments explained.

3. Study Objectives

The primary objective is to assess the clinical benefits of genotype-guided dosing of warfarin against the traditional mode of dosing which uses only clinical factors.

The secondary objective is to prospectively evaluate the accuracy and precision of the pharmacogenetic dose-prediction model used to calculate the maintenance dose for patients in the pharmacogenetics arm [1].

4. Study Design

This is a multicentre, open-label, randomised controlled trial which uses a non-inferiority study design. Patients newly indicated for warfarin therapy will be randomised 1:1 to receive pharmacogenetics-guided or the traditional dosing regimen. The therapeutic International Normalised Ratio (INR) range is considered to be >1.9 and ≤ 3.1.

The disposition of patients who were screened for eligibility will be summarised in a CONSORT flow diagram [2].

5. Sample Size Calculation

The sample size of 270 (135 in each study arm) provides 80% power to detect a non-inferiority margin of 0.5 with a one-sided alpha of 0.05, given a between-group difference in mean number of dose titrations of 1.0 and common standard deviation of 1.4. Primary analysis will use the intention-to-treat population, which will include patients evaluable for the primary endpoint at 12–14 days

Assuming an attrition rate of 15% after randomisation, approximately 320 patients would be enrolled and randomly allocated in a 1:1 fashion to the study groups.

6. Reporting of Patients Baseline Characteristics

Baseline characteristics between the two study groups will be summarised using mean and standard deviation for continuous variables that are normally distributed, or using median and interquartile range if they are skewed. Baseline categorical variables will be described using frequency and percentages. Primary analysis will be carried out based on the principle of intention-to-treat. No interim analyses will be performed. As the study uses a non-inferiority design, a one-sided significance of 5% will be used for statistical comparisons throughout the study.

7. Study Endpoints

7.1 Number of dose titrations performed up to days 12-14

The primary endpoint is the number of dose titrations performed up to 12-14 days following the initiation of warfarin therapy. The average number of dose titration will be compared between study groups using the Student's t-test.

7.2 Secondary Endpoints

7.2.1 Time to stable INR

The time to stable INR is defined as the number of days from randomisation to attaining therapeutic INR for two consecutive measurements that are at least 7 days apart. Patients who were lost to follow-up or did not achieve stable INR during the observation period would be censored at their last follow-up visit. The comparison between study groups will be described using the Kaplan Meier curves and the logrank test. The effect estimate will be quantified using the hazard ratio (HR) and its associated 95% CI.

7.2.2 Number of dose adjustments at 1, 2 and 3 months

The total number of dose adjustments per patient will be tabulated at the end of every month. This endpoint will be analysed using repeated measures analysis via a mixed model with Poisson link function, and will be reported as an incidence rate ratio (IRR).

7.2.3 Number of INR assessments at 1, 2 and 3 months

The total number of INR assessments per patient will be tabulated at the end of every month. The analysis will be performed using a mixed model in the same manner as endpoint 7.2.2.

7.2.4 Proportion of patients whose INR exceeded 3.0

This endpoint will compare the fraction of patients who experienced an INR > 3.0 at any time point while on the trial. The risk ratio and its 95% CI will be used to quantify the effect size between study groups, and the P-value will be calculated using the Chi-square test.

7.2.5 Frequency of bleeding episodes

The number of patients who experienced a bleeding event during the dose-titration phase, which will be classified as major or minor, as described by Levine et al [3] will be tabulated for each study group. The risk ratio and its 95% CI will

be used to quantify the effect size between study groups, and the P-value will be calculated using the Chi-square test.

7.2.6 Incidence of recurrent venous thromboembolism within 90 days of diagnosis

The number of patients who were indicated for warfarin therapy due to venous thromboembolism (VTE) who experienced recurrence within 90 days of diagnosis will be tabulated for each study group. The risk ratio will be used to compare the effect size between study groups, and the P-value will be calculated using the Chi-square test.

7.2.7 Percentage of time spent within therapeutic INR range

The Percentage of Time in Therapeutic Range (PTTR) will be calculated using a linear interpolation method as described by Rosendaal et al (1993) [4]. If the INR value is unavailable at baseline, it will be assumed to be 1.0. The PTTR will be calculated only for patients who received at least 12 days of treatment. Comparison between groups will be performed using a t-test, with data transformations as required.

The endpoint of “Percentage of Time in Therapeutic Range (PTTR)” was introduced by recommendation of the Scientific Review Committee for the **S**urveillance **A**nd **Ph**armacogenomics **I**nitiative for Adverse Drug **RE**actions (SAPhIRE) programme. It was deemed that reporting of this endpoint would facilitate between-trial comparisons with previous studies, and enable meta-analyses of trials evaluating the utility of pharmacogenetics-guided dosing of warfarin.

7.2.8 Accuracy and precision of the pharmacogenetic maintenance dose-prediction model

This endpoint seeks to compare the predicted warfarin maintenance dose vs actual maintenance dose for patients in the pharmacogenetics arm who have achieved stable dose. The accuracy and precision of the model will be evaluated using the mean percentage error (MPE%) and root mean squared error (RMSE%) respectively.

7. References

1. Tham L-S, Goh B-C, Nafziger A et al. A warfarin-dosing model in Asians that uses single-nucleotide polymorphisms in vitamin K epoxide reductase complex and cytochrome P450 2C9. Clin. Pharmacol. Ther. 2006; 80(4):346–55.
2. Schulz KF, Altman DG, Moher D, CONSORT Group. CONSORT 2010 statement: updated guidelines for reporting parallel group randomized trials. Ann. Intern. Med. 2010; 152(11):726–32.
3. Levine M, Gent M, Hirsh J et al. A comparison of low-molecular-weight heparin administered primarily at home with unfractionated heparin administered in the hospital for proximal deep-vein thrombosis. N. Engl. J. Med. 1996; 334(11):677–81.
4. Rosendaal FR, Cannegieter SC, Meer FJM Van Der, Briet E. A Method to Determine the Optimal Intensity of Oral Anticoagulant Therapy. 1993; 69(3):236–239.

Protocol No: PG01/11/06

A Randomized Controlled Trial to Assess the Clinical Benefits of a Pharmacogenetics-Guided Dosing Regimen for Calculating Warfarin Maintenance Dose

Principal Investigator: Dr. Goh Boon Cher[1]
Tel: (65) 6772 4140
Fax: (65) 6777 5545
Email: Boon_Cher_Goh@nuhs.edu.sg

Co-Investigators:

National University Hospital and National University of Singapore:
Dr. Lee Soo Chin[1]
Dr. Andrea Wong[1]
Dr. Michelle Poon[1]
Dr. Liu Te Chih[1]
Dr. Chee Yen Lin[1]
Dr. Peter Robless[2]
Dr. Teoh Hock Luen[3]
Dr. James Yip[4]
Dr Yeo Wee Tiong[4]
Nancy Yong[5]
Prof. Richie Soong[6]
Wu Tuck Seng[7]

Prof. Tai Bee Choo[8]
Dr. Liam R. Brunham[8]

Tan Tock Seng Hospital:
Dr. Tay Jam Chin[9]
Dr. Veerendra Melagireppa Chadachan[9]
Prof. Ponnudurai Kuperan [10]
Ms. Yee May Wong[11]

University of Malaya Medical Centre:
Dr. Bee Ping Chong[12]
Dr. Chen Yeng[12]
Dr. Chee Kok Han[12]
Dr. Jesinda Paul[12]
Dr. Yong Phaik Har[12]

[1]: Department of Haematology-Oncology, National University Health System, Singapore

[2]: Department of Surgery, National University Health System, Singapore
[3]: Department of Medicine, National University Health System, Singapore
[4]: Dept of Cardiology, National University Health System. Singapore
[5]: Department of Pharmacy, National University Health System. Singapore
[6]: Oncology Research Institute, National University of Singapore
[7]: Department of Pharmacy, National University of Singapore
[8]: Yong Loo Lin School of Medicine, National University of Singapore
[9]: Department of General Medicine, Tan Tock Seng Hospital, Singapore
[10]: Department of Haematology, Tan Tock Seng Hospital, Singapore
[11]: Department of Pharmacy, Tan Tock Seng Hospital, Singapore
[12]: Medical Department, Faculty of Medicine, University of Malaya, Jalan University, Kuala Lumpur, Malaysia

Study Sites:

National University Cancer Institute, Singapore
National University Health System
1E Kent Ridge Road
NUHS Tower Block Level 7
Singapore 119228
Telephone: +65 6772-4140
Fax: +65 6777-5545

Tan Tock Seng Hospital
General Medicine II
Tan Tock Seng Hospital
11, Jalan Tan Tock Seng
Singapore 308433
Telephone: +65 6357-7881
Fax: +65 6357-7588

University of Malaya Medical Centre
Jalan University,
50603 Kuala Lumpur
Malaysia
Telephone: +603 7949 2741/2952
Fax: +603- 7954 5682

Table of Contents

Synopsis 4
1. Background 5
2. Rationale and Hypothesis 6
   2.1 Rationale 6
   2.2 Hypothesis 6
3. Patient Selection 6
   3.1 Eligibility Criteria 6
   3.2 Exclusion Criteria 7
4. Investigational Plan 7
   4.1 Patients 7
   4.2 Study Design 7
   4.3 Patient data collection 9
   4.4 Derivation of Warfarin Dosing Model with Validation 9
   4.5 Warfarin Administration 11
   4.6 Genotyping 12
   4.7 Safety 12
   4.8 Potential Risks 12
   4.9 Safety Measures 12
      4.9.1 Safety Reporting to DSRB and HSA 12
   4.10 Potential Benefits 13
   4.11 Trial Administration 13
5. Clinical Significance 13
6. References 14
Appendix 1A 16
Appendix 1B 16
Appendix 2B 16
Appendix 3. 17
Appendix 4

A Randomized Controlled Trial to Assess the Clinical Benefits of a Pharmacogenetics-Guided Dosing Regimen for Calculating Warfarin Maintenance Dose

Synopsis

Background: Interethnic differences in warfarin dose requirements in the Asian population have been well described. Our previous studies showed that warfarin maintenance doses in our multi-ethnic population were closely related to patient demographics and genetic polymorphisms in cytochrome(CYP)P4502C9 and vitamin K epoxide reductase complex subunit 1(VKORC1). A retrospective regression model combining these predictors accounts for 57.8% of the variability in warfarin dose.

Hypothesis: We hypothesize that warfarin dose requirement could be more accurately predicted using a simplified genotyping procedure requiring the identification of a single CYP2C9 allele and a single nucleotide polymorphism of VKORC1 to discern between the 2 major haplotypes H1 and H7.

Aims: The aim is to compare the clinical benefits of genetics-guided dosing versus traditional trial and error dosing with protocol guided-adjustments. Two secondary objectives are (1) to prospectively evaluate a dosing algorithm built on demographics and genetic predictors; (2) to assess the feasibility of a simplified test for CYP2C9*3 and VKORC1 SNP in clinical practice.

Methodology and Statistical Analysis: Details of the statistical design and methodologies for this trial are summarized in the Statistical Analysis Plan (SAP).

Significance: This concerted, multi-disciplinary effort to bring pharmacogenetics-based therapy from bench to bedside has the potential to reduce the efforts incurred with multiple dose titrations of the most commonly prescribed oral anticoagulant. With the aid of mathematical modeling, a simplified and more cost-effective genotyping method could be implementation for the future treatment and prophylaxis of thromboembolic diseases.

1. Background

To date, warfarin remained the most widely prescribed oral anticoagulation agent for long-term prophylaxis and treatment of thromboembolic disorders[1,2]. Since warfarin has a narrow therapeutic index, the discovery of the close association of its dose requirements with genetics polymorphisms in recent years has re-ignited much interest. This drug that has been available since the 1950s is an ideal candidate model for testing the paradigm of pharmacogenetics-based dosing.[3] Previous pharmacogenetics studies showed cytochrome P450 (CYP) 2C9 genotypes to be closely associated with warfarin dose requirements as it undergoes metabolism by this polymorphic enzyme [4-11] The common CYP2C9*1 is considered the wild-type allele while single nucleotide polymorphisms (SNPs) at exon 3, 430C>T for CYP2C9*2 and exon 7, 1075 A>C for CYP2C9*3 are the more common mutants. A recent review by Voora et al summarized the allele frequency of CYP2C9 by ethnic groups.[12] The CYP2C9*1 allele was present in about 80% of the Caucasian population, CYP2C9*2 and CYP2C9*3 accounted for 13% and 7% respectively of the remaining population. The genetic distribution in the Asian population showed predominance of the wild type, a slightly higher distribution of CYP2C9*3 at 18% while CYP2C9*2 was rarely seen. Our previous study showed that CYP2C9*3 was identified in all 3 Asian ethnic groups in Singapore but CYP2C9*2 was not detected in this study comprising 125 patients.[8] Warfarin maintenance was higher for Indians compared to Chinese and Malays. The lowest occurrence of CYP2C9*3 was in Chinese (7%), followed by Malays (11%), then Indians (15%). However, the genetic variants of CYP2C9 alone were insufficient to explain for the difference in maintenance dose requirements between the ethnic groups. More recent findings showed that vitamin K epoxide reductase complex subunit 1 (VKORC1) had a larger impact than CYP2C9 on warfarin dose. This greatly enhanced the predictability of warfarin dose requirements.[13-15] This was confirmed in our local population. VKORC1 variants when assigned to haplotypes based on the method described by Reider, successfully accounted for the difference in warfarin requirements between the Asian ethnic groups that CYP2C9 alone was unable to predict accurately.[13,22] The H1 and H7 haplotypes alone accounted for more than 95% of the haployptes in Chinese and Malays while Indians showed slightly more diversity, with 4 haplotypes (H1, H7, H8 and H9) accounting for 93% of all haplotypes. Therefore, despite the multi-ethnicity, the lack of genetic diversity in the local population allowed VKORC1 genotyping with just one single-nucleotide polymorphism (SNP), at position 381 of the reference sequence (GenBank accession number AY587020), to be sufficient to discern the 2 major haplotypes (H1 and H7). Since the CYP2C9*2 variant was rare in our population, being present in 0%, 1% and 4% in Chinese, Malays and Indians respectively, and the CYP2C9*3 variant was more common being present in 7%, 9% and 8% for Chinese, Malays and Indians respectively, it was safe to “misclassify” the intermediate-metabolizers with the CYP2C9*2 variant with the slow-metabolizers bearing the CYP2C9*3 allele, introducing a deliberate bias toward under-prediction and creating a binary variable for the CYP2C9 predictor.[16]

While it is obvious that demographic, environmental and genetic factors all contribute to the vast inter-individual variability in warfarin dose requirements, traditional dosing algorithms used in guiding its prescribing did not account for the known genetic influence.[17-22] It was in 2005 that the only prospective study validating the clinical utility

of a retrospectively developed pharmacogenetics-guided dosing algorithm using CYP2C9 genotype-guided dosing of warfarin in elective surgical patients showed potential feasibility. It predicted 42% of the variance in maintenance warfarin dose.[19, 23] A study incorporating both CYP2C9 and VKORC1-1639 genotypes further enhanced this application of pharmacogenetics-guided dosing by increasing the accountability of variability in dose to 54.2% and underpredicts daily maintenance requirements in a small validation sample dataset (n = 38) by 0.2 ± 0.8mg/day.[24] Our current model compares favorably with this in terms of accounting for variability in dose ($R^2 = 0.592$), despite using a much less detailed genotyping procedure due to the unique genotypic distribution in our population. This offers a great opportunity to bring pharmacogenetics-guided dosing from bench to bedside.

2. Rationale and Hypothesis

2.1 Rationale

Based on the promising results that genotyping-based strategies for warfarin has shown thus far, a rational approach would be to investigate the possibility of reducing the complexity of the genotyping procedures so as to cut-down the turn around time required for identifying the various genetic variants of CYP2C9 and VKORC1. Timely implementation of a genotype-guided dosing approach in the clinical setting would be required to maximize the potential clinical benefits. In addition, simplified sequencing and genotyping of both CYP2C9 and VKORC1 would pose to be more cost-effective for routine screening in clinical practice.

2.2 Hypothesis and study objectives

We hypothesize that warfarin dose requirement could be more accurately predicted using a simplified genotyping procedure requiring the identification of a single CYP2C9 allele and a single nucleotide polymorphism of VKORC1.

The primary objective is to assess the clinical benefits of genotype-guided dosing of warfarin against the traditional mode of dosing which uses only clinical factors.

The secondary objective is to prospectively evaluate the accuracy and precision of the pharmacogenetic dose-prediction model used to calculate the maintenance dose for patients in the pharmacogenetics arm.

3. Patient Selection

3.1 Eligibility Criteria

1. At least 18 years of age
2. New indication for warfarin therapy
3. No previous history of liver disease; transaminases must be less than 3 times upper limit of normal and bilirubin within normal range

4. No previous history of malabsorption syndrome or chronic diarrheal conditions
5. Written, informed consent

3.2 Exclusion Criteria

1. Uncontrolled hypertension
2. Peptic ulcer disease
3. Any other medical conditions as deemed unfit for warfarin therapy based on clinical judgement of primary physician

4. Investigational Plan

4.1 Patients

This is a randomized, controlled, open-label trial targeted at accruing 320 patients with new indication for warfarin therapy. 10mls of blood will be taken from all the patients once informed consent has been taken. For patients randomized to the pharmacogenetics-guided dosing group, this 10mls of blood will be immediately sent for genotyping studies. Genotyping results will be available for pharmacogenetics-guided dosing within 3 working days, (ranging 3 to 5 days). During this period, if patients need to be initiated on anticoagulation, a low molecular weight heparin, Fraxiparine, (*other suitable alternatives of LMWH may be used instead) will be given. Fraxiparine will be overlapped with warfarin for 2 to 3 days until target INR is achieved. Elective cases should have the pharmacogenetics-based warfarin dose available at the time of warfarin therapy. For patients randomized to the traditional dosing regime, the blood will be stored and genotyped retrospectively at the end of the study. Overlapping of warfarin with Fraxiparine or heparin till target INR is achieved is allowed for this group as per normal clinical practice. All warfarin dosage adjustments based on INR results will be according to the current protocol used by the NUH Anticoagulant Clinic (see Appendix 2). INR will be checked within 7 days following every change in dose. In addition to the INR obtained from the laboratory, we will also use a single drop of blood from the venepuncture to determine the INR with the CoaguCheck XS system (Roche). The INR obtained from the CoaguCheck system will, however, not influence the warfarin dosing.

4.2 Study Design

Study endpoints and Statistical Considerations

Refer to Appendix 4 – Statistical Analysis Plan

Accrual of study subjects with new indication for warfarin therapy (n = 350)

↓

Informed consent

↓

Patient Randomization; Determination of target therapeutic INR

Traditional dosing regimen Pharmacogenetics-guided dosing

Dose Initiation Regimen (first 3 days)		
	**Traditional dosing regimen**	**Pharmacogenetics-guided dosing**
Day 1	Low-molecular weight heparin	Low-molecular weight heparin
Day 2	Low-molecular weight heparin	Low-molecular weight heparin
Day 3	Low-molecular weight heparin<br>Warfarin 5mg	Low-molecular weight heparin<br>Warfarin tailored dose (if pharmacogenetics results available)
Day 4	Low-molecular weight heparin<br>Warfarin 4mg if age >75 years<br>All others 5mg	Low-molecular weight heparin<br>Warfarin tailored dose (if pharmacogenetics results available)
Day 5	Low-molecular weight heparin<br>Warfarin 3mg	Low-molecular weight heparin<br>Warfarin tailored dose (if pharmacogenetics results available)<br>Switch to standard protocol if pharmacogenetics results not available by day 5
Day 6	Low-molecular weight heparin<br>Check INR<br>Warfarin dose according to Appendix 1A<br>For all subsequent INRs checked during dose initiation phase, Warfarin dose will be determined by Appendix 1B	Low-molecular weight heparin<br>Warfarin tailored dose (if pharmacogenetics results available)<br>Check INR if three doses of warfarin had been administered, and dose subsequent warfarin dose according to Appendix 2A.<br>For all subsequent INRs checked during dose initiation phase, Warfarin dose will be determined by Appendix 2B
7-9 days after initiation of warfarin	INR check	INR check
12-14 days after initiation of warfarin	INR check	INR check

* Once therapeutic INR is achieved, low-molecular heparin should be stopped, and INR should be re-checked at least once within a week (this may coincide with the INR check on day 7-9 or day 12-14)

Maintenance Dose Regimen

- Once INR is maintained within the target range of 2-3 for 2 or more consecutive checks without a change in dose for at least 3 months, patients are considered to have reached maintenance dose.
- The total period of follow up for this study is approximately 3 months
- The number and frequency of visits should be between 3 to 5 during the first 2 weeks of initiating therapy although more visits may be required depending on the individual patient's response to warfarin therapy.

4.3 Patient data collection

Baseline characteristics will be obtained from all patients accrued into the study. These include race, age, sex, weight, height, liver and renal function, and concomitant medications. The INR values and dates of tests for each patient will also be recorded, together with any tests of the prothrombin time (PT), partial thromboplastin time (PTT). Each adjustment in warfarin dose will also be recorded and the corresponding INR checked within 1 week. Patients potentially eligible for this study will be screened from both the inpatient and outpatient services. INR will be performed using the standard venous blood sample.

4.4 Derivation of Warfarin Dosing Model with Validation

A total of 220 complete datasets were available for constructing the warfarin dose requirement linear regression model. Demographic predictors used for model-building included age, weight, sex, ethnic group (Chinese, Malay, Indian, Others), serum albumin concentration, INR and the presence of CYP2C9*3 variant and VKOR-381 CC genotypes. Log-transformation was applied to warfarin doses to achieve normality in its distribution. Univariate anaylses were conducted to retain the closely associated predictors for further model building. Only those variables with $p < 0.1$ in the initial analysis were considered as sufficiently associated with warfarin dose and retained for further model-building. Only those predictors showing statistical significance of $p < 0.05$ and did not demonstrate collinearity were retained in the final model. A data-splitting method that randomly assigned approximately 50% of the available dataset for model building and the remaining half for model validation was employed.

One hundred and eleven patients were randomly selected into the model-building dataset and the remaining 109 formed the validation cohort. The initial multiple linear regression model using the logarithmic derivation of warfarin dose featured the patient age, body weight, Indian and Chinese ethnic groups as well as the pharmacogenetic predictors *VKORC1-381* CC, *VKORC1-381* TC, *CYP2C9*2* and *CYP2C9*3* as predictors. It exhibited an r^2 of 0.609. Collinearity checks of the model showed tolerance values of between 0.287 for *VKORC1-381* CC genotype to 0.898 for age, and corresponding VIF values of 3.49 to 1.114. However, the p values of the 2 ethnic group and *CYP2C9*2* predictors did not reach statistical significance ($p>0.05$). Hence, they were removed from the final regression model. Details of the final linear model are available on request. Standardized residuals were between -2.9 to 2.0. Overall, this model showed only a very slight compromise in correlation (0.78 to 0.77) when compared to the initial model and still explained for 59.2% of the variation in warfarin dose.

Data from remaining subjects (n = 109) were used to validate this derived model. It showed an overall under-prediction of 0.37 ± 1.26 mg/day, ranging from -5.1 to 2.4 mg/day.

Figure 1. Difference in warfarin dose against average of actual and estimated doses, with 95% limits if agreement (broken line) and regression line (solid line). Subjects with *VKORC1-381* CC genotype are marked by the open circles, TC genotype by solid triangles and TT genotypes by open squares. ($r^2 = 0.346$)

Predicted minus actual dose (mg/day)

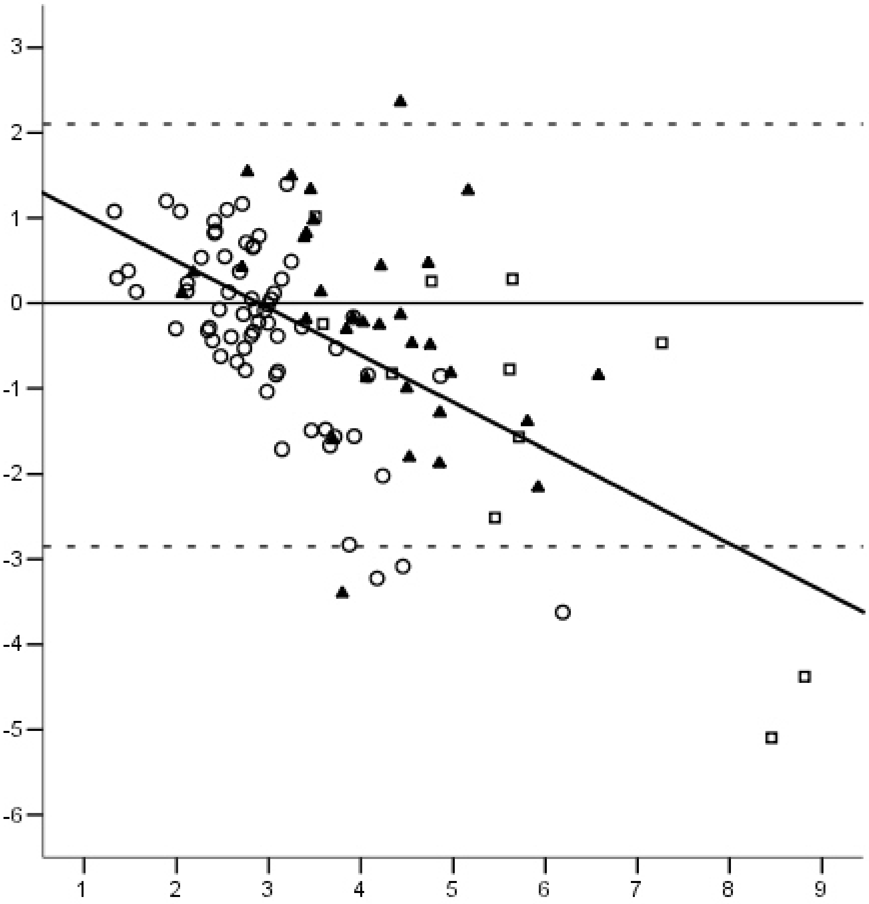

Average warfarin dose (mg/day)

4.5 Warfarin Administration

All predicted warfarin dose will be administered by rounding down to the nearest 0.5 mg. Warfarin (Marevan®) is available as 1mg (brown), 3 mg (blue) and 5 mg (pink) oral tablets from GlaxoSmithKline Pte. Ltd. Patients and/or their caregivers will be given dietary advice upon initiation of warfarin therapy to avoid drug-food interactions affecting warfarin pharmacokinetics.

Suitable alternatives of warfarin or generic drugs may be used instead.

4.6 Genotyping

Details of genotyping were described in our previous papers for CYP2C9 and VKORC1 respectively.[8,26] Genotyping tests for CYP2C9 will be reduced to a single variant, testing only for CYP2C9*3 and VKORC1 to one SNP -381. Samples consisting of 10 mls of blood will be drawn from patients once written, informed consent is given. All samples will be labeled and coded in identification numbers to ensure patient confidentiality.

4.7 Safety

Investigators are responsible for monitoring the safety of subjects who have entered this study and for alerting the Principal Investigator of any event that seems unusual, even if this event may be considered an unanticipated benefit to the subject.

The investigator is responsible for appropriate medical care of subjects during the study.

The investigator remains responsible for following, through an appropriate health care option, adverse events that are serious or that caused the subject to discontinue before completing the study. The subject should be followed until the event resolves or is explained. Frequency of follow-up is left to the discretion of the investigator.

4.8 Potential Risks

Patients in the pharmacogenetics-guided dosing group in this study should not be subjected to any potential risks greater than those in the traditional dosing cohort or not participating. INR will be checked within 3 to 5 days of the first dose of warfarin and within 1 week for every dose adjustment. The dose will be immediately adjusted according to Appendices 1 (for traditional arm dosing) and 2 (for pharmacogenetics arm dosing) if any deviation from the target INR is observed. The only risks involved in this study are the blood draws, which carry a very small risk of local pain, bleeding and infection. However, as the blood draw for the pharmacokinetics study will be done at the same time as the routine laboratory tests (eg INR) after the patient has reached a stable INR, the patients are not put at any higher risk than those not participating.

4.9 Safety Measures

Safety measures that will be used in the study include physical examinations and clinical laboratory tests (haematology and blood chemistries).

4.9.1 Safety Reporting to DSRB and HSA

An adverse event is any untoward medical occurrence in a clinical investigation patient administered a pharmaceutical product and which does not necessarily have to have a causal relationship with this treatment. A Serious Adverse Event is any untoward medical occurrence at any dose that:

1. Results in death or;
2. Is life-threatening or;
3. Requires inpatient hospitalization or prolongation of existing hospitalization or;
4. Results in persistent or significant disability/incapacity or;

5. Is a congenital anomaly/birth defect;
6. Is a medically important event.

All SAEs that are unexpected and related to the study drug will be reported. The investigator is responsible for informing HSA no later than 15 calendar days after first knowledge that the case qualifies for expedited reporting. Follow-up information will be actively sought and submitted as it becomes available. For fatal or life-threatening cases, HSA will be notified as soon as possible but no later than 7 calendar days after first knowledge that a case qualifies, followed by a complete report within 8 additional calendar days.

4.10 Potential Benefits

Patients will not get any monetary gains from this study. In the event that a blood test needs to be repeated, patients will receive transport reimbursement.

4.11 Trial Administration

Patient records will be filed in the Cancer Therapeutics and Research Group Office, National University Hospital in the form of Case Record Forms (CRFs). The investigator must record all serious adverse events, treatment failures, regardless of treatment or relationship to study drug, as soon as he/she is informed of the event. Investigators must report immediately to the Principal Investigator any serious adverse events. All SAE will be reported to the Domain-Specific Research Board (DSRB) and the Medical Clinical Research Committee (MCRC), Ministry of Health in accordance to published guidelines. Any serious and or unexpected adverse event should be medically well documented and the information made available as soon as possible.

5. Clinical Significance

This concerted, multi-disciplinary effort to bring pharmacogenetics-based therapy from bench to bedside has the potential to reduce the efforts incurred with multiple dose titrations of the most commonly prescribed oral anticoagulant. With the aid of mathematical modeling, a simplified and more cost-effective genotyping method could be implemented as a routine for the future treatment and prophylaxis of thromboembolic diseases. If successful, this would be one of the first models of prospective application of pharmacogenetics from bench to bedside. This is of particular importance to Asians, as the model of dosing used here is derived from and hence applicable only to the Asian patients. This information also has the potential to affect the dosing requirements of other common medications that are metabolized by the same enzyme, eg tolbutamide.

6. References

1. The topic 20 generic drugs by units in 2004. Drug Topics, 7 Mar 2005; http://www.drugtopics.com/drugtopics/data/articlestandard/drugtopics/102005/150069/article.pdf
2. Hirsh J, Dalen JE, Anderson DR, Poller L, Bussey H, Ansell J, Deykin D, Brandt JT. Oral anticoagulants: mechanism of action, clinical effectiveness, and optimal therapeutic range. Chest 1998;114(5 Suppl):445S-469S.
3. Hayes WJ Jr. Control of Norway rats with residual rodenticide warfarin. Public Health Rep. 1950;24;65(47):1537-55.
4. Kamali F, Khan TI, King BP, Frearson R, Kesteven P, Wood P, Daly AK, Wynne H. Contribution of age, body size, and CYP2C9 genotype to anticoagulant response to warfarin. Clin Pharmacol Ther. 2004;75(3):204-12.
5. Scordo MG, Pengo V, Spina E, Dahl ML, Gusella M, Padrini R. Influence of CYP2C9 and CYP2C19 genetic polymorphisms on warfarin maintenance dose and metabolic clearance. Clin Pharmacol Ther. 2002;72(6):702-10.
6. Taube J, Halsall D, Baglin T. Influence of cytochrome P-450 CYP2C9 polymorphisms on warfarin sensitivity and risk of over-anticoagulation in patients on long-term treatment. Blood. 2000;96(5):1816-9.
7. Takahashi H, Wilkinson GR, Padrini R, Echizen H. CYP2C9 and oral anticoagulation therapy with acenocoumarol and warfarin: similarities yet differences. Clin Pharmacol Ther. 2004;75(5):376-80.
8. Zhao F, Loke C, Rankin SC, Guo JY, Lee HS, Wu TS, Tan T, Liu TC, Lu WL, Lim YT, Zhang Q, Goh BC, Lee SC. Novel CYP2C9 genetic variants in Asian subjects and their influence on maintenance warfarin dose. Clin Pharmacol Ther. 2004;76(3):210-9.
9. Hillman MA, Wilke RA, Yale SH, Vidaillet HJ, Caldwell MD, Glurich I, Berg RL, Schmelzer J, Burmester JK. A prospective, randomized pilot trial of model-based warfarin dose initiation using CYP2C9 genotype and clinical data. Clin Med Res. 2005;3(3):137-45.
10. Veenstra DL, Blough DK, Higashi MK, Farin FM, Srinouanprachan S, Rieder MJ, Rettie AE. CYP2C9 haplotype structure in European American warfarin patients and association with clinical outcomes. Clin Pharmacol Ther. 2005;77(5):353-64.
11. Takahashi H, Wilkinson GR, Caraco Y, Muszkat M, Kim RB, Kashima T, Kimura S, Echizen H. Population differences in S-warfarin metabolism between CYP2C9 genotype-matched Caucasian and Japanese patients. Clin Pharmacol Ther. 2003;73(3):253-63.
12. Voora D, McLeod HL, Eby C, Gage BF. The pharmacogenetics of coumarin therapy. Pharmacogenomics. 2005;6(5):503-513.
13. Rieder MJ, Reiner AP, Gage BF, Nickerson DA, Eby CS, McLeod HL, Blough DK, Thummel KE, Veenstra DL, Rettie AE. Effect of VKORC1 haplotypes on transcriptional regulation and warfarin dose. N Engl J Med. 2005;352(22):2285-93.
14. Wadelius M, Chen LY, Downes K, Ghori J, Hunt S, Eriksson N, Wallerman O, Melhus H, Wadelius C, Bentley D, Deloukas P. Common VKORC1 and GGCX

polymorphisms associated with warfarin dose. Pharmacogenomics J. 2005;5(4):262-70.
15. Veenstra DL, You JH, Rieder MJ, Farin FM, Wilkerson HW, Blough DK, Cheng G, Rettie AE. Association of Vitamin K epoxide reductase complex 1 (VKORC1) variants with warfarin dose in a Hong Kong Chinese patient population. Pharmacogenet Genomics. 2005;15(10):687-91.
16. Takahashi H, Kashima T, Nomoto S, Iwade K, Tainaka H, Shimizu T, Nomizo Y, Muramoto N, Kimura S, Echizen H. Comparisons between in-vitro and in-vivo metabolism of (S)-warfarin: catalytic activities of cDNA-expressed CYP2C9, its Leu359 variant and their mixture versus unbound clearance in patients with the corresponding CYP2C9 genotypes. Pharmacogenetics. 1998;8(5):365-73.
17. Crowther MA, Ginsberg JB, Kearon C, Harrison L, Johnson J, Massicotte MP, Hirsh J. A randomized trial comparing 5-mg and 10-mg warfarin loading doses. Arch Intern Med. 1999;159(1):46-8.
18. Kovacs MJ, Rodger M, Anderson DR, Morrow B, Kells G, Kovacs J, Boyle E, Wells PS. Comparison of 10-mg and 5-mg warfarin initiation nomograms together with low-molecular-weight heparin for outpatient treatment of acute venous thromboembolism. A randomized, double-blind, controlled trial. Ann Intern Med. 2003;138(9):714-9.
19. Voora D, Eby C, Linder MW, Milligan PE, Bukaveckas BL, McLeod HL, Maloney W, Clohisy J, Burnett RS, Grosso L, Gatchel SK, Gage BF. Prospective dosing of warfarin based on cytochrome P-450 2C9 genotype. Thromb Haemost. 2005 ;93(4):700-5.
20. Sconce EA, Khan TI, Wynne HA, Avery P, Monkhouse L, King BP, Wood P, Kesteven P, Daly AK, Kamali F. The impact of CYP2C9 and VKORC1 genetic polymorphism and patient characteristics upon warfarin dose requirements: proposal for a new dosing regimen. Blood. 2005;106(7):2329-33.
21. Levine M, Gent M, Hirsh J, Leclerc J, Anderson D, Weitz J, Ginsberg J, Turpie AG, Demers C, Kovacs M. A comparison of low-molecular-weight heparin administered primarily at home with unfractionated heparin administered in the hospital for proximal deep-vein thrombosis. N Engl J Med. 1996;334(11):677-81.
22. Lee SC, Ng SS, Oldenburg J, Chong PY, Rost S, Guo JY, Yap HL, Rankin SC, Khor HB, Ye TC, Ng KS, Soong R, Goh BC. Inter-ethnic variability of warfarin maintenance requirement is explained by VKORC1 genotype in as Asian population. Manuscript in press, Clin Pharmacol Ther.
23. Henne KR, Gaedigk A, Gupta G, Leeder JS, Rettie AE. Chiral phase analysis of warfarin enantiomers in patient plasma in relation to CYP2C9 genotype. J Chromatogr B Biomed Sci Appl. 1998;710(1-2):143-8.

Appendix 1A

**INR**	**Warfarin Dose**	**LMWH**	**Remarks**
<1.5	4mg	Continue	Recheck INR in 1-3 days[c]
1.51-3	3mg	Stop if INR≥2	Recheck INR within 1 week[c]
3.01-3.5	2mg	Stop	Recheck INR within 1 week[c]
>3.5	Omit	Stop	Recheck INR in 1-3 days[c]

Appendix 1B

**INR**	**Actions**
<1.5	Increase warfarin dose by 25% (round down to nearest 0.5mg). Re-check INR the next day
1.51-3	Maintain same warfarin dose
3.01-3.5	Reduce warfarin dose by 25% (round **down** to nearest 0.5mg)
3.51-4	Reduce warfarin dose by 50% (round **down** to the nearest 0.5mg) Re-check INR the next day
>4	Omit dose for the day. Re-check INR the next day

Appendix 2A**INR**	**Warfarin Dose**	**LMWH**	**Remarks**
<1.5	Increase warfarin dose by 25% (round **down** to nearest 0.5mg).	Continue	Recheck INR in 1-3 days[c] and refer to Appendix 2B for subsequent dosing.
1.51-3	Maintain same warfarin dose	Stop if INR≥2	Recheck INR within 1 week[c] and refer to Appendix 2B for subsequent dosing.
3.01-3.5	Reduce warfarin dose by 25% (round **down** to nearest 0.5mg)	Stop	Recheck INR within1 week[c] and refer to Appendix 2B for subsequent dosing.
>3.5	Omit dose for the day	Stop	Recheck INR in 1-3 days[c] and restart warfarin at 75% of original dose (ie. reduce warfarin dose by 25 % rounded down to nearest 0.5mg). Refer to Appendix 2B for subsequent dosing

Appendix 2B

**INR**	**Actions**
<1.5	Increase warfarin dose by 25% (round **down** to nearest 0.5mg). Re-check INR the next day
1.51-3	Maintain same warfarin dose
3.01-3.5	Reduce warfarin dose by 25% (round **down** to nearest 0.5mg)
3.51-4	Reduce warfarin dose by 50% (round **down** to the nearest 0.5mg) Re-check INR the next day
>4	Omit dose for the day. Re-check INR the next day

Appendix 3.

Warfarin Dosing Algorithm for ACC Harmonised Clinic during maintenance phase

Target INR: 2.0-3.0 by Protime

If INR <2 or >3, venous INR should be obtained as the correct INR value

INR	ACTION
< 1.5	Increase dosage by 1mg / dose;<br>repeat blood test for INR in 7 days<br>NB. If patient has prosthetic heart valve, inform doctor-in-charge immediately*
1.5-1.9	Increase dosage by 0.5mg / dose;<br>repeat blood test for INR in 7 - 10 days
2.0-3.0	No change
3.1-3.4	No bleeding :<br>Repeat INR within 2 weeks if INR is in range on same dose previously for more than 6 months and patient does not have obvious bleeding risks, otherwise:<br>- Decrease dose by 0.5mg
3.5-4.0	No bleeding: decrease dosage by 0.5mg / dose;<br>repeat blood test for INR in 7 days<br>Bleeding: inform doctor-in-charge of patient immediately*
4.1-5.0	No bleeding: stop warfarin for 2 days;<br>decrease dosage by 1mg / dose;<br>repeat blood test for INR in 5 - 7 days<br>Bleeding: inform doctor-in-charge of patient immediately*
5.1-5.5	No bleeding: stop warfarin for 3 days;<br>repeat blood test for INR in 3-5 days;<br>restart warfarin if INR is in therapeutic range (decrease dosage by 1.5mg);<br>Bleeding: inform doctor-in-charge immediately*
> 5.5	Stop warfarin;<br>inform doctor-in-charge immediately*

INR	Variation	Assessment Date
Out of range<br>(refer to Appendix 3- Warfarin Dosing Algorithm)	-	3-10 days
Within target range	≥ 0.8 units<br>< 0.8 units	4-6 weeks<br>3 months

NB. A repeat blood test should be performed 1 week to 10 days later for all NEW patients (i.e. first-timers) to check the INR so that the dose may be confirmed, even if the initial INR after discharge is within target range.

Appendix 4 Statistical Analysis Plan
