# Supplementary material for: Genotype-guided versus traditional clinical dosing of warfarin in patients of Asian ancestry: a randomized controlled trial
Source: BMC Med. 2018 Jul 10;16:104. doi: 10.1186/s12916-018-1093-8 (PMC6038204; doi:10.1186/s12916-018-1093-8)
Supplement: Supplementary file 2 — Table S1. Baseline characteristics of patients excluded from primary analysis. (DOCX 25 kb) [file 12916_2018_1093_MOESM2_ESM.docx]

| **Table S1. Baseline characteristics of patients excluded from primary analysis** | | |
| --- | --- | --- |
|  | **Traditional dosing**  **(n = 27)** | **Genotype-guided dosing**  **(n = 26)** |
| Age, mean (SD), y | 59.5 (14.0) | 60.3 (14.9) |
| Weight, mean (SD), kg | 60.4 (14.6) | 64.2 (14.1) |
| Race, No. | | |
| - Chinese | 18/27 | 16/26 |
| - Malay | 4/27 | 4/26 |
| - Indian | 3/27 | 1/26 |
| - Others | 2/27 | 5/26 |
| *CYP2C9* genotype, No./total | | |
| - Presence of *3 allele | 1/26 | 2/26 |
| *VKORC1* -381 genotype, No./total | | |
| - C/C | 9/27 | 19/26 |
| - C/T | 13/27 | 6/26 |
| - T/T | 5/27 | 1/26 |
| Indication, No./total |  |  |
| - Atrial fibrillation | 3/26 | 12/24 |
| - Stroke | 2/26 | 1/24 |
| - Deep vein thrombosis | 9/26 | 5/24 |
| - Pulmonary embolism | 2/26 | 3/24 |
| - Left ventricular thrombus | 3/26 | 1/24 |
| - Others | 5/26 | 4/24 |
| Amiodarone, No./total | 0/26 | 3/24 |
| LMWH, No./total | 12/26 | 8/25 |
| Medical history, No./total |  |  |
| - Stroke | 1/26 | 3/25 |
| - Deep vein thrombosis | 3/26 | 0/25 |
| - Pulmonary embolism | 1/26 | 0/25 |
| - Myocardial infarction | 1/26 | 4/25 |
| - Congestive heart failure | 2/26 | 3/25 |
| - Hypertension | 12/26 | 14/25 |
| Type 2 diabetes mellitus | 9/26 | 10/25 |
|  | | |
